# Effect of Preoperative Mastoid Ventilation on Tympanoplasty Success

**DOI:** 10.1155/2014/169123

**Published:** 2014-10-12

**Authors:** Mehmet Metin, Zeynep Kizilkaya Kaptan, Sedat Dogan, Hasmet Yazici, Cem Bayraktar, Hakan Gocmen, Etem Erdal Samim

**Affiliations:** ^1^Hendek State Hospital, 54300 Sakarya, Turkey; ^2^Ankara Research and Training Hospital, Ministry of Health, 06340 Ankara, Turkey; ^3^Faculty of Medicine, Adiyaman University, Ear Nose Throat Clinic, 02200 Adiyaman, Turkey; ^4^Faculty of Medicine, Balikesir University, Ear Nose Throat Clinic, 10145 Balikesir, Turkey

## Abstract

*Purpose*. This study was conducted with the aim of investigating the relationship between mastoid air cell volumes and graft success after tympanoplasty. *Material and Methods*. This study was performed retrospectively with patients undergoing type I tympanoplasty and antrostomy. A total of 57 patients (20–35.09% female and 37–64.91% male) with a mean age of 29.69 ± SD (range 12–56 years) were included in the study. The patients were invited for a control at the 1st, 3rd, and 12th months, and otoscopic examinations and audiometric tests were performed. The temporal bone computed tomography images were screened with the 4800 Dpi optic resolution scanner and transferred to the computer environment in JPG format in order to calculate the mastoid air cell volume, and the volumes were calculated using the Autocad 2007 program. *Results*. Although, the graft success was determined to be better in the well-ventilated group, no significant difference could be found between the groups in terms of graft success at the 1st, 3rd, and 12th months (*P* > 0.05). No statistically significant difference could be found between the three groups in terms of the preoperative and postoperative hearing gains (*P* > 0.05).

## 1. Introduction

Chronic otitis media (COM) is a common problem. Genetic and environmental factors affect the progression of otitis media to chronicity. A decrease in the mastoid air cells has been shown to be related with atelectatic ear diseases, cholesteatoma, and chronic otitis media with effusion [1, 2]. Whether poorly developed mastoid cells are the reason or a result of otitis media has not been proven yet. Whether mastoid cell ventilation affects the outcomes of tympanoplasty is an issue which has not been discussed sufficiently and on which only a limited number of studies have been performed. The effect of preoperative mastoid ventilation on tympanoplasty results is controversial.

In this study, the relationship between the mastoid air cell volumes and the graft success after tympanoplasty was investigated. The relationships between the preoperative mastoid air cell ventilation and the obtained hearing gains, air-bone gaps, and ventilation degrees according to gender and the patient ages were also investigated.

## 2. Material and Methods

Patients who had been diagnosed with chronic otitis media for whom surgery had been planned, and on whom temporal bone computed tomographies had been performed, were included in the study. Patients who had undergone otoscopy, microscopy, audiometry, and computed tomography tests and who had undergone type I tympanoplasty and antrostomy were included in the study. Patients who were determined to have cholesteatoma intraoperatively, revision cases, and patients whose follow-up findings were missing were excluded from the study. Patients underwent type 1 tympanoplasty and surgeries are done by the same surgeon and the same operative technique is used. Only antrostomy was performed following a postauricular incision (as pathology was not detected in the other cells). Patients whose ossicles were intact and mobile were included in the study. Patients who were found to have encountered complications such as bleeding and infection were excluded from the study. Temporal muscle fascia was used as graft in all the patients. On the control visits, otoscopy examinations and audiometry tests were performed and the results were recorded. Of the 57 patients who met the criteria of the study, 20 (35.09%) were male and 37 (64.91%) were female, with a mean age of 29.69 (range 12–56).

Temporal bone computed tomographies were performed using a Hitachi-Pronto AR HP spiral scanner. The tomography sections were 2 mm in thickness and parallel to the orbitomeatal line. The temporal bone computed tomography images were scanned using the 4800 Dpi optic resolution scanner and transferred to the computer environment in JPG format in order to calculate the mastoid bone volume using the Cavalieri technique. The mastoid volumes of CT images which had been transferred to the computer environment were calculated using the Autocad 2007 program. On CT, the air cell area in the mastoid bone on the operated side was calculated and the bony part of the mastoid was not included in this calculation. Air cells containing soft tissue areas in which inflammatory activity was present were not included in the volume calculation of the air cells. The patients were divided into three groups as the poorly ventilated group, the moderately ventilated group, and the well ventilated group according to the mastoid air cell ventilations detected on the preoperatively performed temporal bone CT.

## 3. Statistical Analysis and Results

The data were analyzed using the SPSS for Windows 11.5 package program. Whether the distribution of constant variables was near normal or not was analyzed using the Shapiro Wilk test. The descriptive statistics were shown as mean ± standard deviation for constant variables, and the nominal variables were shown as numbers and percentages (%). The presence or absence of a significant change between the air-bone gap levels in the preoperative period and at the postoperative 12th month was evaluated using the Dependent *t*-test. The nominal variables were analyzed using the Pearson's Chi-square test. A *P* level of <0.05 was accepted as statistically significant.

*Mastoid Volume*. The total mastoid cell ventilations were divided to three groups as the result of the measurements was made according to the Cavalieri principle, benefiting from the preoperative temporal CT (Table 1): Group I: ventilated, Group II: moderately ventilated, and Group III: poorly ventilated (Table 1). The average mastoid volume of all the patients was measured as 3.8486 cm³ (range 0.7765 cm³–14.891 cm³). The mean volume was 11.7322 cm³ for Group I, 7.1755 cm³ for Group II, and 2.1237 cm³ for Group III. Statistical analyses were performed between the mastoid ventilation groups and the graft success (Table 2).

Graft success was analyzed at the 1st, the 3rd, and the 12th months in the mastoid cell groups. In Group I, the graft success in 7 patients was 85.71% at the 1st, the 3rd, and the 12th month. In Group II, the graft success in 14 patients was 85.71% (12/14 patients) at the 1st month, 78.57% (11/14 patients) at the 3rd Month, and 78.57% (11/14 patients) at the 12th month. In Group III, the graft success of 36 patients was 75% (27/36 patients) at the 1st month, 69.44% (25/36 patients) at the 3rd month, and 63.88% (23/36 patients) at the 12th month.

No statistically significant difference was determined between the mastoid ventilation groups in terms of graft success rates at the 1st, the 3rd, and the 12th months (*P* = 0.352).

*Audiometry Results*. In the preoperative audiometries of the patients, the mean threshold values of the side which would be operated and the mean threshold values of the air conduction and bone conduction thresholds at the 1st, 3rd, and 12th months were measured (Table 3).

Statistical analyses including air-bone gaps and air conduction gains were performed between the mastoid ventilation groups (Table 3).

A statistically significant decrease was found between the mean air-bone gaps and the air conduction thresholds at the postoperative 12th month compared to the preoperative values (*P* < 0.001). However there was no significant difference between ventilation groups in terms of audiometry results at the end of the 12th month (*P* > 0.05). 

*Age and Gender Groups*. 20 (35%) of the patients were male and 37 (65%) were female, with a mean age of 19.69 (range 12–56). The mean age of the patients was 24.14 years in Group I, 25.57 years in Group II, and 29.27 years in Group III. No statistically significant difference was found between the gender groups in terms of graft success rates at the end of the 12th month (*P* = 0.217). 

*Operated Side*. The right ears of 27 patients and the left ears of 30 patients were operated.

No statistically significant difference was determined between the right and the left sides in terms of graft success rates at the end of the 12th month (*P* = 0.234).

## 4. Discussion

The mastoid air cell system has a great importance in middle ear physiology. Tumarkin and Holmquist claimed that mastoid cells provided an air reservoir for the middle ear and demonstrated that they played a role in the pressure regulation of the middle ear [3, 4]. This hypothesis was also supported by Sade and Fuchs [2]. Frisberg et al. was the first who analyzed the relationship between the mastoid air cell size and the prognosis of middle ear disease [5]. Holmquist and Bergstroem also studied that issue later. Holmquist and Bergstroem showed that the success of middle ear surgery depended on mastoid cell ventilation. In his study, Holmquist and Bergstroem measured the mastoid volumes using Schüller X-Rays performed preoperatively. They showed that middle ear retraction was greater in patients who had undergone tympanomastoidectomy compared to those who had undergone tympanoplasty without mastoidectomy [6]. Therefore, they advocated that well ventilated mastoid cells should not be intervened during surgery [6]. Bonding suggested that the reason for unsuccessful tympanomastoidectomy in children depended on the mastoid cell system [7]. However, Siedentop, Palva, and Gimenez did not find such a relationship in the studies they carried out with 63, 61, and 52 chronic otitis media patients, respectively [8–10]. In the study of Onur et al. carried out with 255 patients, they observed that the graft success obtained in ears with diploic mastoiditis was more favorable compared to the pneumatic ones and they concluded that there was no relationship between the mastoid ventilation amount and the myringoplasty success [11]. The authors used Schüller X-Ray as the imaging method for measurement of the mastoid volume. In our study, mastoid volumes were evaluated as three-dimensional through calculating the mastoid volumes using high resolution computed tomography and Autocad. As the Schüller X-Ray provides a two-dimensional imaging, the technique used in our study may provide more precise results. Moreover previous studies authors [2, 6, 11] investigated effect of mastoid ventilation on tympanoplasty success but they performed mastoidectomy in their studies but it is known that mastoidectomy decreases mastoid volume and affects middle ear pressure. Based on this opinion some authors studied regeneration of mastoid air cells. Kanemaru et al. reported that regeneration of mastoid air cells (MACs) can effectively eliminate intractable COM [12–14]. In another study, Kanemaru et al. investigated the ability of regenerated MACs to restore gas exchange function and contribute to the improvement of eustachian tube function and indicated that tissue-engineered regeneration of MACs improves eustachian tube function and gas exchange in the middle ear [15]. In our study all the patients had undergone only antrostomy without mastoidectomy and relationship between preoperative mastoid volume and tympanoplasty success is evaluated.

The results of studies about mastoid ventilation are controversial. Holmquist and Bonding carried out studies indicating that mastoid ventilation affected the surgical results [7, 16]; however, there are also researchers who did not detect this relationship [8–11]. Similar results were obtained also in our study. Although we assessed better results in well-ventilated group there was no statistically significant difference between the well-ventilated group and the poorly ventilated group in terms of graft success. Furthermore, no statistically significant differences were determined between the mastoid cell ventilation and the postoperative airway gains.

## 5. Conclusion

In conclusion, no statistically significant relationship could be found between the preoperative mastoid cell ventilation and the postoperative graft success in patients who had undergone only antrostomy together with tympanoplasty as chronic otitis surgery. In addition, no statistically significant difference could be determined between the mastoid ventilation and the postoperative hearing gains.

## Figures and Tables

**Table 1:** 

	Mastoid volume (cm³)	Patient number	%	Mean volume
Group I	10 cm³ and above	7	12.28%	11.7322 cm³
Group II	Between 5 and 10 cm³	14	24.56%	7.1755 cm³
Group III	Between 0 and 5 cm³	36	69.91%	2.1237 cm³

**Table 2:** Graft success rates of the mastoid ventilation groups at the 12th month.

Mastoid ventilation	Graft success
Group I (*n* = 7)	6 (85.7%)
Group II (*n* = 14)	11 (78.6%)
Group III (*n* = 36)	23 (63.9%)
*P* ^†^	0.352

^†^Pearson Chi-square test.

**Table 3:** Preoperative and postoperative hearing threshold averages and air-bone gaps measured in all the patients and the mastoid ventilation groups.

	Preoperative			1st month			3rd month			12th month		
	ac	bc	abg	ac	bc	abg	ac	bc	abg	ac	bc	abg
	(dB)	(dB)	(dB)	(db)	(dB)	(dB)	(dB)	(dB)	(dB)	(dB)	(dB)	(dB)
Patients	48	22	26	36.3	15.6	20.6	32.3	15.9	16.4	30.8	15.3	15.5
Group I	39.71	16.85	22.86	34.4	13	21.4	26.1	13	13.1	22.9	11.9	11
Group II	45.14	24.35	20.79	35.3	18.3	17	31.1	17.1	13.9	31.1	17.6	13.4
Group III	46.69	21.86	24.83	37.2	17.6	19.5	34.2	18.1	16.1	32.8	17.6	17.6

ac: air way conduction; bc: bone conduction; abg: air bone gap.
